# Supplementary material for: Evaluating soil salinity dynamics under drip irrigation in the Manas River Basin, Xinjiang: a long-term analysis (1996–2019)
Source: PeerJ. 2025 Apr 28;13:e19295. doi: 10.7717/peerj.19295 (PMC12045282; doi:10.7717/peerj.19295)
Supplement: Supplemental Information 1 [file peerj-13-19295-s001.docx]

Sheet 1 heading and tabs

**Chinese Translated in English**

总-全部数据 All Data

Sheet 2 heading and tabs

jm-建模数据 JM Modeling Data

序号 S.No

经度 longitude

纬度 latitude

盐分 salinity

盐分1 Salt content 1

Sheet 3 heading and tabs

yz-验证数据 Yz - Verify data

序号 S.No

经度 Longitude

纬度 Latitude

盐分 Salinity
